# Strengthening of CYFRA 21-1 using urine creatinine correction as potential endometriosis biomarker 

**DOI:** 10.12688/f1000research.135167.2

**Published:** 2025-05-14

**Authors:** Nicko Pisceski Kusika Saputra, Samsulhadi Samsulhadi, Hendy Hendarto, Ashon Sa’adi, Widodo J Pudjirahardjo, I Wayan Arsana, Relly Yanuari, Sri Ratna Dwiningsih

**Affiliations:** 1 Fertility and Reproductive Endocrinology Division, Department of Obstetrics and Gynecology, Faculty of Medicine, Universitas Airlangga, Surabaya, East Java, Indonesia; 2 Department of Obstetrics and Gynecology, Faculty of Medicine, Universitas Riau, Pekanbaru, Riau, Indonesia; 3 Department of Public Health, Faculty of Medicine, Universitas Airlangga, Surabaya, East Java, Indonesia; 4 Fertility and Endocrinology Division, Department of Obstetrics and Gynecology, Faculty of Medicine, Universitas Brawijaya, Malang, East Java, Indonesia

**Keywords:** Endometriosis, CYFRA 21-1, CYFRA/Cr, Urine Creatinine, Proliferative phase.

## Abstract

**Background:**

This study aimed to determine the accuracy of CYFRA 21-1 using urine creatinine correction (CYFRA/Cr) as a biomarker of endometriosis.

**Methods:**

This study includes 73 patients from the Indonesian population, with 38 endometriosis and 35 non-endometriosis patients based on laparoscopy. Urine detection of CYFRA 21-1 was done by ELISA method and corrected by urine creatinine constant factor (CYFRA/Cr). Urine creatinine us detected using the ECLIA method.

**Results:**

The CYFRA/Cr ratio was identified in the proliferative and secretory phases. CYFRA 21-1 and CYFRA/Cr levels were significantly higher in endometriosis and were higher in the proliferative phase compared to the secretory phase. The best accuracy was obtained in CYFRA which was corrected with urine creatinine in the proliferative phase with a sensitivity value, specificity, and cutoff value of 94.7%, 94.4%, and of 3,547.99 ng/gr, respectively, compared to CYFRA 21-1 urine levels without correction of creatinine.

**Conclusions:**

The CYFRA to creatinine urine ratio detected in the proliferative phase showed the optimum sensitivity and specificity compared to CYFRA 21-1 spot urine. It has the potential to be a biomarker of endometriosis.

## Introduction

The diagnosis of endometriosis is reported to be delayed to almost 7–10 years from disease onset because menstrual pain is often considered normal ( Hogg & Vyas, 2015). Laparoscopy is the gold standard for the diagnosis of endometriosis ( Tokushige *et al.*, 2011). Laparoscopy has 90.1% (95% CI 81.0-95.1) sensitivity, 40% specificity (95% CI 23.4-59.3), PPV 81,0% (95% CI 71.0-88.1), NPV 58.8% (95% CI 36.0-78.4) with 77.1% accuracy (95% CI 67.7-84.4). Even though it has high sensitivity and PPV with low specifity and NPV, diagnosing endometriosis with laparoscopy and histopathology can give accurate diagnosis ( Gratton et al., 2022). Compared with histopathology, laparoscopy gave a sensitivity and specificity of 97.68% and 79.23%, respectively, in the diagnosis of endometriosis ( de Almeida Filho *et al.*, 2008). In another study, sensitivity was 94% (95% CI: 80%–98%), and specificity was 79% (95% CI: 67%–87%) ( Wykes *et al.*, 2004). However, laparoscopic surgery is an invasive procedure that sometimes provides unwanted complications and higher costs, causing a delay in the diagnosis and management of endometriosis ( Tokushige *et al.*, 2011).

Biomarker detection as a diagnosis is very interesting and can advance the management of infertility and pelvic pain. Previous studies have identified endometriosis biomarkers from various samples, including blood, cervical mucus, and urine ( Fassbender *et al.*, 2015). Diagnosis with biomarkers is very promising because it is not invasive, relatively inexpensive, and relatively fast compared to surgery. Urine is a potential specimen in the diagnosis of endometriosis, especially urine biomarkers.

In 2011, the protein that was found in the urine of endometriosis patients was identified as a cytokeratin-19 fragment, namely, CYFRA 21-1 but the sample size was small (n=16) ( Tokushige *et al.*, 2011). Cytokeratin-19 is found in simple and complex squamous cell epithelium, so it is commonly found in basal cells and squamous membranes. Cytokeratin-19 is a low molecular weight cytokeratin (LMWCK) ( Jose *et al.*, 2013). It is an acidic cytokeratin with a molecular weight of 40 kDa and is also expressed in glandular-type epithelium, one of which is the endometrial gland epithelium ( Schweizer *et al.*, 2006). Although the mechanism of excretion of cytokeratin-19 fragments (CYFRA 21-1) in the urine is not clear, the presence of impaired renal filtration which is characterized by increasing serum creatinine levels that cause decreasing in the level of cytokeratin-19 fragments (CYFRA 21-1) in the urine accompanied the increasing its serum level ( Kashiwabara *et al.*, 1998). The mean levels of cytokeratin-19 fragments (CYFRA 21-1) in the urine of women with endometriosis were significantly higher in the proliferative phase than in the secretory phase. The sensitivity value in the proliferative phase is 94.12%, with a cutoff value of 4 ng/ml/g creatinine, while the sensitivity value in the secretory phase is 31.5% ( Gjavotchanoff, 2015). However, CYFRA 21-1 as a biomarker is still inconclusive, so further study is needed to confirm this finding ( Liu *et al.*, 2015), with a bigger sample size. The inconsistency of results may be due to unstable CYFRA 21-1 and heavily influenced by temperature, menstrual cycle, and fluctuating levels.

Protein that is excreted into the urine is very volatile. Therefore, the description of spot urine protein levels can automatically describe the actual condition. When examining, routine urine protein levels must be compared with something relatively stable in the urine ( Kamińska *et al.*, 2020). The levels of cytokeratin-19 fragments (CYFRA 21-1) are circadian ( Gjavotchanoff, 2015; Nolen *et al.*, 2015). Urine creatinine is a determinant and correction factor that can be used because its levels are relatively stable in the urine; thus, the results obtained are not influenced by variations in fluid intake. This becomes the background for measuring spot urine protein levels. The standard for measuring urine protein is 24-h urine protein due to fluctuating protein excretion. However, the 24-h urine collection has many problems, so an alternative method can be used, i.e., the urine protein and creatinine ratio recommended by the National Kidney Foundation and Kidney Disease Outcomes Global Improving (KDIGO) ( National Kidney Foundation, 2002). Muscle metabolism leads to the irreversible dehydration of body creatine and creatine phosphate and creates creatinine as a waste product. More than 90% of the creatinine is accumulated in skeletal muscle. The formation rate of this product is constant; 2% of body creatine is converted to creatinine every 24 hours. However, the older the individual, the slower the rate ( Barr *et al.*, 2005).

The inconsistency of study results needs to be reanalyzed to strengthen the potential of CYFRA 21-1 as a biomarker of endometriosis by using correction of urine creatinine to urine CYFRA (CYFRA/Cr). Therefore, this study aimed to analyze the accuracy of CYFRA 21-1 through urine creatinine correction for the diagnosis of endometriosis. Analysis was also carried out based on menstrual cycle.

## Methods

### Ethics and consent considerations

This study has passed the ethical review of the Medical and Health Research Ethics Unit of the Faculty of Medicine, Universitas Riau with register number B/091/UN19.5.1.1.8/UEPKK/2021. It was declared to be ethically appropriate to seven WHO 2011 standards. The date of the certificate was 10 ^th^ September 2021.

We confirm that we have obtained permission to use the data from the patients included in this presentation. Each patient gave written and verbal informed consent after they have been informed about the research’s objective, what was sampled and its procedure, as well as the provided data usage in this research. The patients also knew that this research would be published but no identifiable data from the patients would be included in the publication.

### Study design

This is an analytic study with a cross-sectional design. The study was conducted at the Arifin Ahmad General Hospital, Provinsi Riau, from October 2019 to October 2021. Examination of cytokeratin-19 fragment (CYFRA 21-1) levels in urine was conducted at the Integrated Biomedical Laboratory, Faculty of Medicine, Universitas Riau, Indonesia. Examination of urine creatinine levels was conducted at the Prodia Laboratory Pekanbaru, Indonesia.

### Sample selection

This study included all patients from the Fertility Clinic of Arifin Ahmad General Hospital with indications of laparoscopic surgery. The patients were approached directly when they were visiting in-person to the clinic and categorized based on the inclusion and exclusion criteria. Subjects were recruited by several steps. Pre-sampling from the patients with endometriosis indication based on the symptom and ultrasonography analysis. Endometriosis degree was determined based on the laparoscopy analysis based on rASRM and based on histopathology assessment of the sample. The sampled tissue was based on the lesion; if it was a cyst, then the whole cyst was taken, if it was implant, then as much as the lesion was taken from the sample. The inclusion criteria were age of 30–40 years, normal body mass index (BMI) of 18.5–24.9 kg/m ^2^, and menstrual cycle of 26–38 days. Age and BMI information was procured from anamnesis and direct observation when the patients were in the clinic. The patient’s menstrual cycle was obtained during the observation at the clinic in the form of Last day of menstruation, duration of menstruation within the last 3 months and confirmed during the menstruation day after laparoscopy and post-operation control. The menstrual cycle information was then analysed for its proliferation and secretion phase. The exclusion criteria were patients taking hormonal drugs such as contraception, other medications such as anti-inflammatory drugs, and antioxidant drugs and patients currently suffering from lung cancer, gastrointestinal cancer, urinary tract infections (based on the results of medical records, chest X-ray, urine routine examination, liver function), impaired kidney function (based on glomerular filtration rate (GFR), creatinine examination), and diabetes mellitus. A total of 73 patients were selected based on laparoscopy, with 38 endometriosis patients and 35 non-endometriosis patients. The control of this study was the laparoscopy patient, participants of contraception program from National Population and Family Planning Board. Non-endometriosis laparoscopy patients were those undergoing surgery for septum uterus repair, tube repair and IUD translocation with adjusted characteristic to be similar with case group.

### Urine CYFRA 21-1 levels test

The ELISA method was used to examine the urine CYFRA 21-1 level. Urine was collected before laparoscopy and stored on ice or refrigerator for no more than 2 h before processing. During transport to the laboratory, the cold chain must be well maintained; hence the samples were transported using an icebox. The collected urine was stored in a refrigerator at −80°C before the examination. The urine used was uncentrifuged urine. The ELISA kit used is the Human Cytokeratin 19 ELISA Kit (product number RAB1409).

Standard serial dilution was performed to obtain the following concentrations of CYFRA 21-1: 0.061 ng/ml, 0.154 ng/ml, 0.384 ng/ml, 0.960 ng/ml, 2.4 ng/ml, 6 ng/ml, 15 ng/ml, and blank. All reagents and samples were brought to room temperature (18–25°C) before use. Examination was performed in duplicate. Approximately 100 ul of each standard and sample was added to the well. The wells were covered and incubated for one night at a temperature of 4°C, followed by washing. Approximately 100 ul antibody was added to each well and incubated for 1 h at room temperature, mixed thoroughly, and washed. Approximately 100 ul of streptavidin was added and incubated for 45 min followed by washing. Approximately 100 ul of TMB substrate was added to each well, 50 ul of stop solution was added. The absorbance value was read at a wavelength of 450 nm. Standard plotting was performed to determine the equation on the standard curve.

### Urine creatinine and urine CYFRA/Cr ratio test

Urine creatinine is a constant determinant in determining urine cytokeratin-12 levels, so the ratio of cytokeratin and urine creatinine can be identified. The ECLIA method was used to examine the urine creatinine level at the Prodia Pekanbaru Laboratory. The ratio of urine cytokeratin-19 fragment (CYFRA 21-1) levels to creatinine (CYFRA/Cr) was calculated by comparing the levels of cytokeratin-19 fragments (CYFRA21-1) to urine creatinine levels in ng/g units and the assessment was done twice.

### Data analysis

All data were processed using IBM SPSS Statistics 28.0. Numerical data was tested for data normality using the Kolmogorov–Smirnov and Shapiro–Wilk tests. Patient characteristics were analyzed using T-test and the Mann–Whitney test. Sensitivity and specificity tests for urinary cytokeratin-19 (CYFRA 21-1) and CYFRA/Cr levels were analyzed using the ROC test. The AUC values and coordinates of the curve were transformed into Microsoft Excel, and the data was then entered into a graph line with a marker to obtain the intersection point or cutoff point value of sensitivity and specificity.

## Results

Table 1 presents the characteristics of the study subjects, consisting of age, BMI, and kidney function based on serum creatinine and GFR values. As presented in Table 1, no differences were found in patient characteristics analyzed by cycle phase in both endometriosis and non-endometriosis cases. Based on serum creatinine and GFR data, it was found that all study subjects were in the normal range, which means that kidney function was in good condition. Therefore, the bias toward the excretion of cytokeratin-19 fragments (CYFRA 21-1) was negligible. Based on the results of chest X-ray examination in all study subjects, the heart and lungs were within normal limits. Based on examination of blood sugar, liver function, and routine urine, all samples were found to be within normal limits so they could be included.

**Table 1.** Patient characteristics.

Characteristics	N	Cycle phase	Mean ± SD	Median (Min–Max)	p
**Non-endometriosis **					
Age (years)	18	Proliferation	35.33 ± 2.701		0.968
	17	Secretion	35.29 ± 3.037		
BMI (kg/m ^2^)	18	Proliferation		23.71 (19.20–24.30)	0.843
	17	Secretion		23.70 (21.60–24.50)	
GFR (ml/min/1.73 m)	18	Proliferation	114.444 ± 6.697		0.666
	17	Secretion	115.294 ± 4.580		
Serum creatinine (mg/dl)	18	Proliferation		0.65 (0.65–0.80)	1
	17	Secretion		0.70 (0.60–0.70)	
**Endometriosis**					
Age (years)	19	Proliferation		35 (30–39)	0.055
	19	Secretion		32 (30–39)	
BMI (kg/m ^2^)	19	Proliferation		23.63 (20.90–24.30)	0.174
	19	Secretion		23.2 (18.40–24.40)	
Serum creatinine (mg/dl)	19	Proliferation		0.7 (0.60–0.80)	
	19	Secretion		0.6 (0.60–0.70)	0.068
GFR (ml/min/1.73 m)	19	Proliferation		113 (95–123)	0.109
	19	Secretion		116 (109–123)	

### Levels of cytokeratin-19 fragments (CYFRA 21-1) spot urine

**Figure 1.**
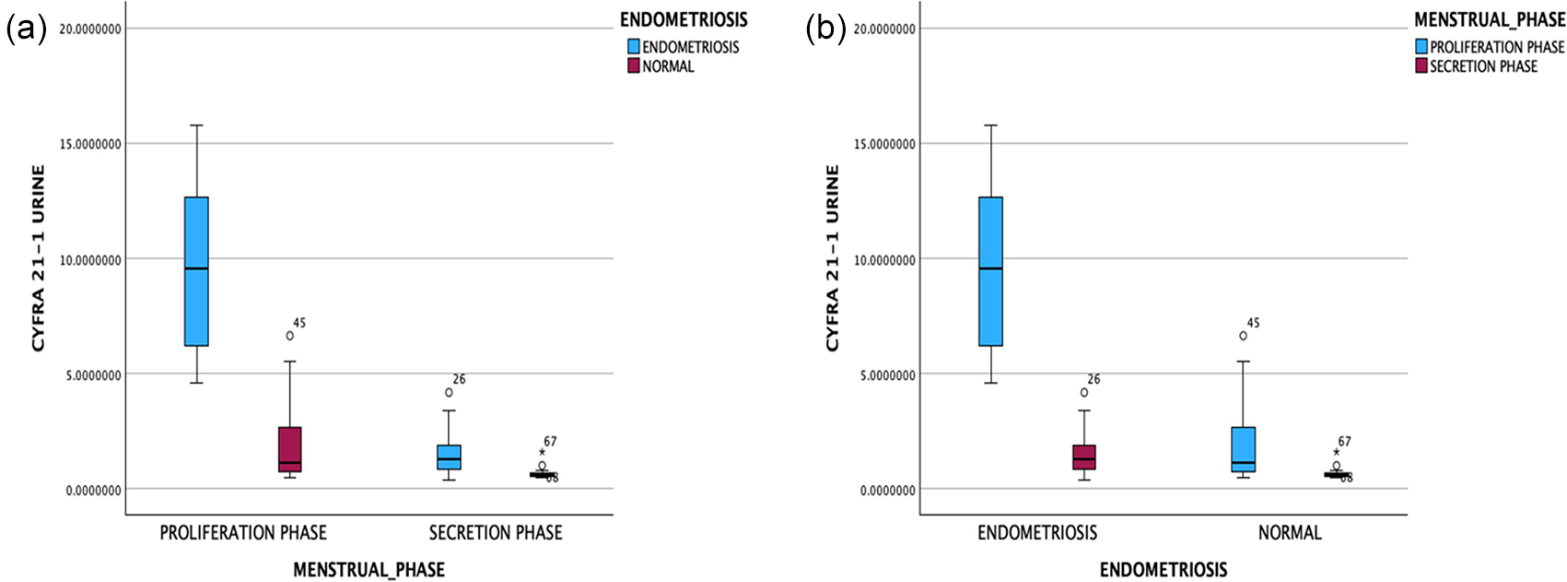
Levels pf cytokeratin-19 fragments (CYFRA 21-1) spot urine based on endometriosis incidence (a) and menstrual cycle phase (b).

**Figure 2.**
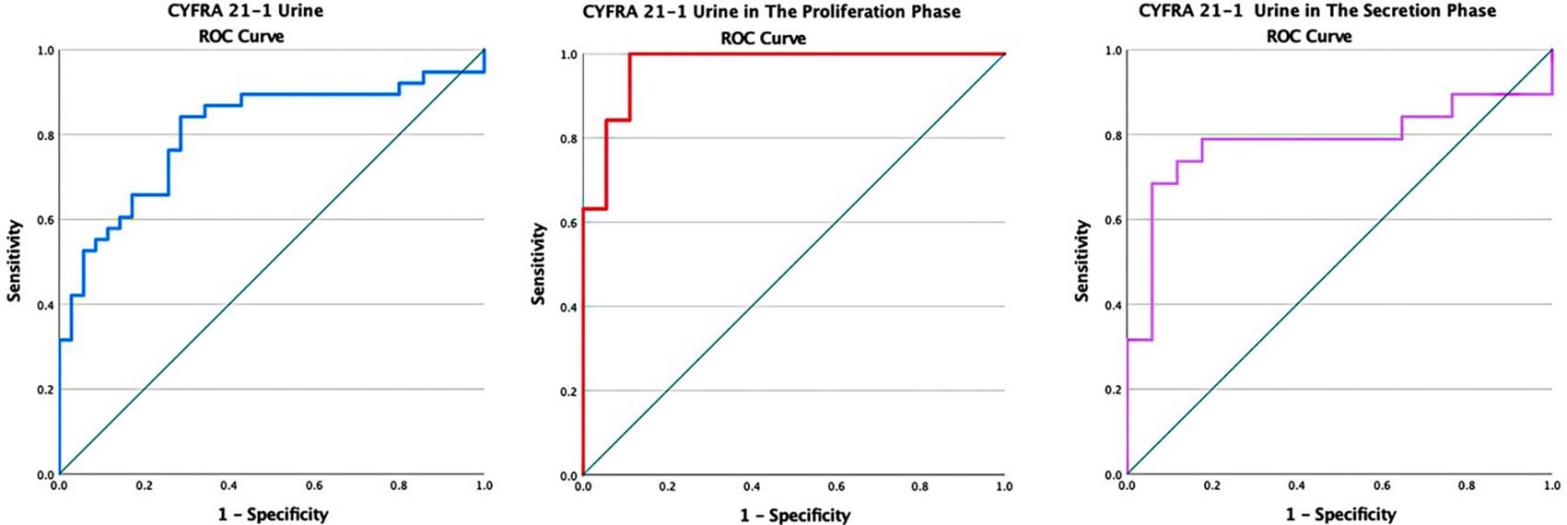
ROC curve of CYFRA 21-1 urine in endometriosis without distinction of the menstrual cycle, in the proliferative, and secretory phase.

Figure 1 presents the levels of cytokeratin-19 fragments (CYFRA 21-1) urine. It can be seen that the levels of cytokeratin-19 fragments (CYFRA 21-1) were significantly higher in the endometriosis group than in the non-endometriosis group (p < 0.05). The levels of cytokeratin-19 fragments (CYFRA 21-1) were significantly higher in the proliferative phase compared to the secretory phase in both the endometriosis and non-endometriosis groups. The AUC area at 0.81 (95% CI 0.70-0.91), p = 0.00, sensitivity of 76.3% and a specificity of 74.3% with the cutoff point of the cytokeratin-19 fragment (CYFRA 21-1) was 1.26 ng/ml ( Figure 2).

In the analysis based on the cycle phase, it was found that the AUC in the proliferative phase was 0.97 (95% CI: 0.92-1). The considerable interpretation of the AUC for more than 90% was very good, with a p-value of <0.05 (p = 0.000). Meanwhile, the AUC in the secretory phase was 0.78 (95% CI: 0.62-0.95). The interpretation of the AUC is classified as moderate with a significance of p = 0.000. In the proliferative phase, the sensitivity and specificity were 89.5% and 88.9%, respectively, with a cutoff value of 5.024 ng/ml for the levels of cytokeratin-19 fragments (CYFRA 21-1). At the same time, in the secretory phase, the sensitivity and specificity were 78.9% and 82.4%, respectively, with a cutoff of 0.77 ng/ml.

### Cytokeratin-19 fragment (CYFRA 21-1) levels to creatinine (CYFRA/Cr) urine ratio

**Figure 3.**
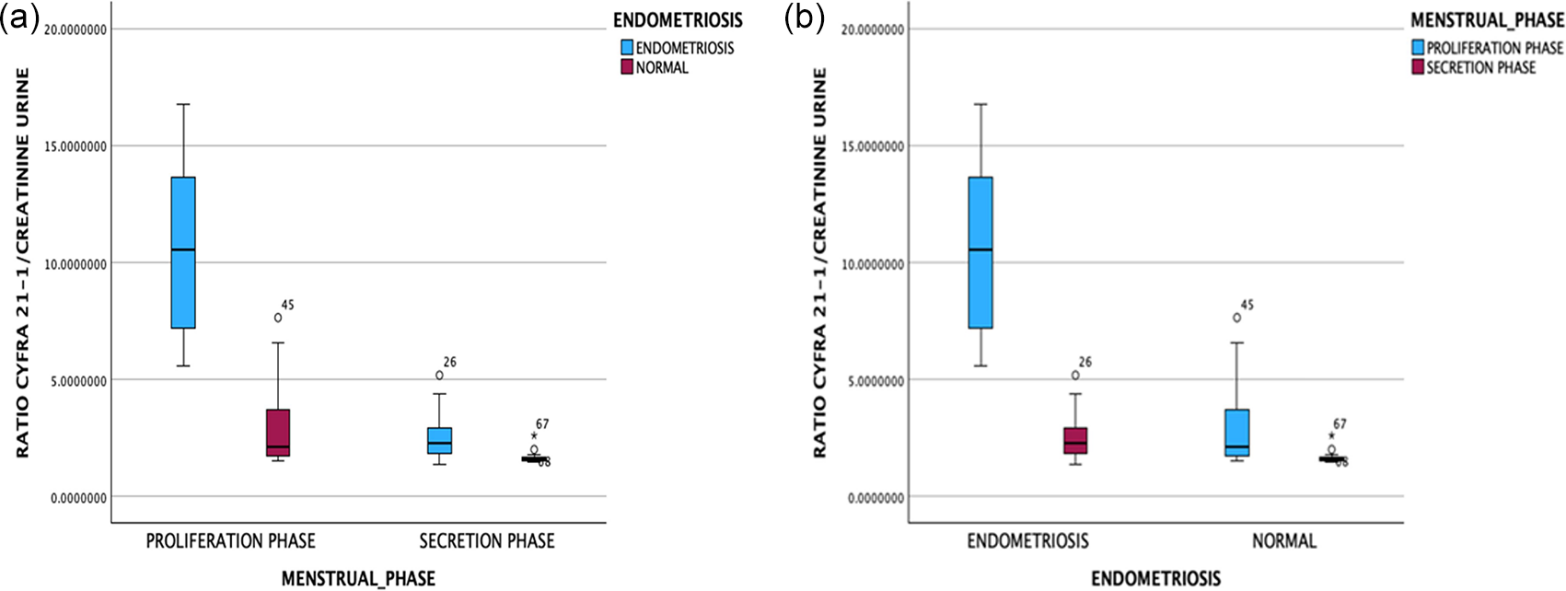
Cytokeratin-19 fragment (CYFRA 21-1) levels to creatinine (CYFRA/Cr) urine ratio based on endometriosis incidence (a) and menstrual cycle phase (b).

Fluctuating random urine cytokeratin-19 fragment (CYFRA 21-1) levels need to be corrected with urine creatinine as a constant factor and generate a CYFRA 21-1 urine creatinine ratio or CYFRA/Cr. As presented in Figure 3, the cytokeratin-19 fragment (CYFRA 21-1) levels to creatinine (CYFRA/Cr) urine ratio was significantly higher in the endometriosis group than the non-endometriotic group (p < 0.05). Moreover, the cytokeratin-19 fragment (CYFRA 21-1) levels to creatinine (CYFRA/Cr) urine ratio was significantly higher in the proliferative phase compared with the secretory phase in both the endometriosis and non-endometriosis groups.

**Figure 4.**
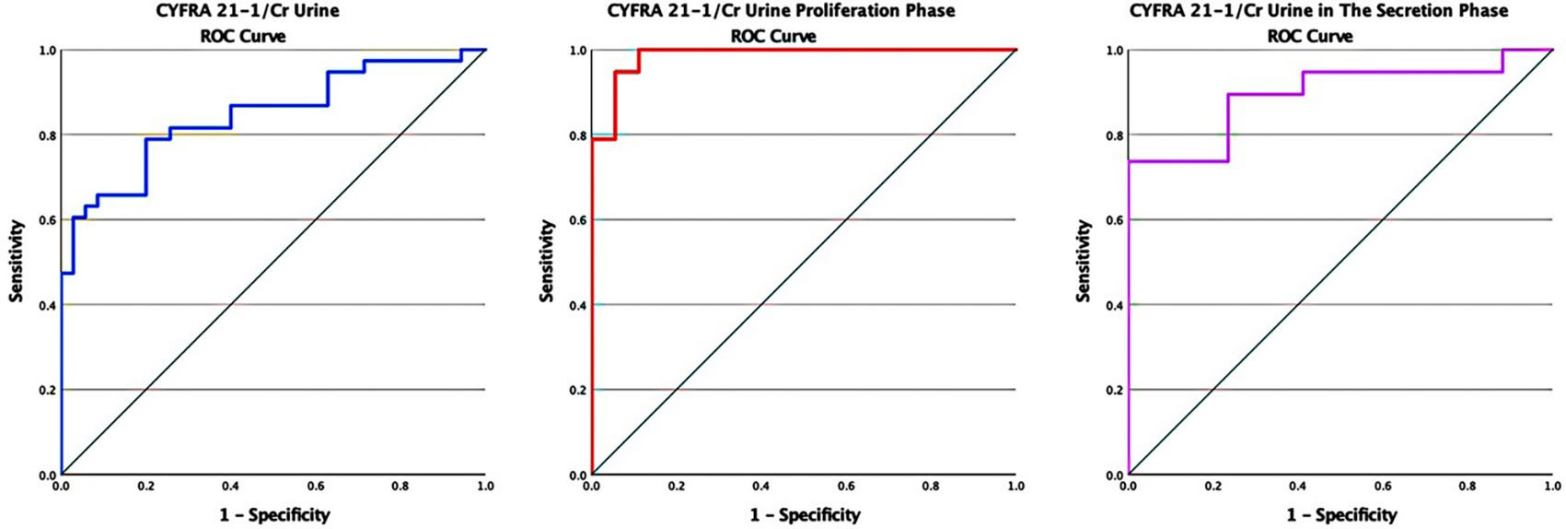
ROC curve of CYFRA/Cr urine in endometriosis without distinction of the menstrual cycle in the proliferative and secretory phase.

The AUC was 0.85 (95% CI: 0.76-0.94). The interpretation of an AUC of more than 0.8 is good, with p < 0.05 (p = 0.00). The sensitivity and specificity were 78.9% and 77.1%, respectively, with a cutoff value of 1,647.11 ng/gr in the cytokeratin-19 fragment (CYFRA 21-1) levels to creatinine (CYFRA/Cr) ratio ( Figure 4).

In the analysis based on the menstrual cycle phase, which was obtained based on the ROC curve in the proliferative phase, the AUC was 0.99 (95% CI: 0.96-1). The interpretation of an AUC of more than 0.9 was considered very good, with p < 0.05 (p = 0.015). The AUC in the secretory phase was 0.90 (95% CI: 0.79-1), and the interpretation of the AUC was classified as moderate with insignificant meaning p = 0.056. In the proliferative phase, the sensitivity and specificity were 94.7% and 94.4%, respectively, with a cutoff value of 3,547.99 ng/gr in the cytokeratin-19 fragment (CYFRA 21-1) level to creatinine (CYFRA/Cr) urine ratio. This result has the best sensitivity and specificity. At the same time, in the secretory phase, the sensitivity and specificity were 73.7% and 76.5%, respectively, with a cutoff value of 657.15 ng/gr.

## Discussion

This research found that The CYFRA/Cr ratio was identified in the proliferative and secretory phases. CYFRA 21-1 and CYFRA/Cr levels were significantly higher in endometriosis and were higher in the proliferative phase compared to the secretory phase. The best accuracy was obtained in CYFRA which was corrected with urine creatinine in the proliferative phase with a sensitivity value, specificity, and cutoff value of 94.7%, 94.4%, and of 3,547.99 ng/gr, respectively, compared to CYFRA 21-1 urine levels without correction of creatinine.

In this study, patient characteristics include age, BMI, and renal function based on serum creatinine values, and GFR values are important variables to avoid bias. Age included in inclusion criteria is in the range of 30–40 years due to the possibility that age will affect the production of cytokeratin-19 fragments (CYFRA 21-1). Renal function is also influenced by age ( National Kidney Foundation, 2002). Continuous decrease in GFR from a mean 123 mL/min/1.73 m ^2^ at age 20–29 years to a mean 65 mL/min/1.73 m ^2^ at age 80–89 years is a decline of about 10 mL/min per decade of age ( Delanaye & Rule, 2015). The presence of impaired renal function leads to the inability of the kidney to clear cytokeratin-19 fragments (CYFRA 21-1), resulting in increased levels of cytokeratin-19 fragments in the blood ( Rastel *et al.*, 1994).

Other confounding factors identified were routine chest X-rays and routine urine examinations. Based on chest X-ray examination, it was within normal limits. Routine urine examination is a screening examination to rule out other diseases that affect the results, i.e., urinary tract infections, urinary tract stones, or urinary tract cancer. X-ray examination is important to avoid bias in the presence of other diseases that can increase the production of cytokeratin-19 fragments (CYFRA 21-1), i.e., lung cancer.

Endometriosis is a disorder with various pathophysiologies. Hormonal, genetic, immunological, and oxidative stress factors are thought to be involved in the complex pathophysiology of endometriosis. This study found the high activity of oxidative stress in endometriotic tissue damage ( Chen *et al.*, 2020).

The level of cytokeratin-19 fragment (CYFRA 21-1) spot urine in women with endometriosis was significantly higher than those without endometriosis. Cytokeratin-19 is expressed in glandular-type epithelium, one of which is the endometrial gland. Cytokeratin-19 normally occurs in the glandular epithelium both in the proliferative, secretory, and atrophic phases of the endometrium based on IHC examination. There is a more consistent expression in the functional layer, whereas the basal zone is usually focally stained. In the proliferative epithelium, cytokeratin-19 was seen in the basal and apical cytoplasm ( Stewart *et al.*, 2011).

Ectopic endometrial tissue causes an immune hyper-reaction that affects the breakdown of cytokeratin-19 to CYFRA 21-1 ( Gjavotchanoff, 2015). Systemic release of cytokeratin-19 can occur through several mechanisms, including cellular apoptosis, abnormal mitosis, or release due to cell proliferation. Extracellular release occurs at the intermediate stage of epithelial cell apoptosis and during cell damage ( Fujita *et al.*, 2004).

An increase in M2 activity was seen based on the pathophysiology of endometriosis through macrophage activity. Macrophages are supposed to act as classical phagocytes but, instead, increase their proliferation to form a neoplastic transformation. In this process, ectopic endometrial cells that bind to macrophages are thought to be damaged and release intracellular proteins ( Capobianco & Rovere-Querini, 2013).

Cytokeratin-19 formed the smallest molecular weight of 40 kDa and consists of 400 amino acids encoded by the KRT 19 gene. The cytokeratin-19 fragment (CYFRA 21-1) formed soluble protein with a small molecular weight of 30 kDa (less than 19 kDa) as the molecule can pass through the glomerular filtration and be excreted in the urine ( Jose *et al.*, 2013). These results strengthen the findings of Tokushige *et al.* (2011), who stressed urine protein is detected in endometriosis, which is then detected as CYFRA 21-1 through proteomic and western blotting techniques. At that time, Tokushige *et al.* (2011) could not determine how CYFRA 21-1 is released into the urine. Of note, the urine used in the previous study was urine that had been centrifuged. This study is also in line with the results obtained in the Gjavotchanoff (2015), but the difference is in the correction of the creatinine factor.

Cytokeratin-19 fragment (CYFRA 21-1) is a microprotein that breaks down easily at room temperature. In a study on the stability of the levels of cytokeratin-19 fragments (CYFRA 21-1) in urine at room temperature, it showed a decrease in levels associated with time ( Nisman *et al.*, 2002). Therefore, to maintain its stability after sample collection, it should be stored in a refrigerator. In this research, it was stored at −80°C. To maintain cold chain protein in the urine, the transportation process used an icebox. In a recent study in 2019 from a population in Korea, no significant difference was observed in levels of cytokeratin-19 fragments (CYFRA 21-1) between endometriosis and non-endometriosis. Meanwhile, the samples examined in this study were serum, and endometriosis controls were cases of non-endometrial ovarian tumors ( Cho & Kyung, 2019).

A difference was observed in the levels of cytokeratin-19 fragments (CYFRA 21-1) between centrifuged and non-centrifuged urine, and the best results were obtained from uncentrifuged urine ( Gjavotchanoff, 2015; Nisman *et al.*, 2002). In this study, urine that was not centrifuged was used, although at the time of sample preparation the researchers prepared two preparations, i.e., centrifuged and non-centrifuged. During optimization, the best concentration was obtained from non-centrifuged urine. Inconsistency of results can also be caused by CYFRA 21-1 is a fluctuating protein so that spot urine test becomes inaccurate.

If the levels of cytokeratin-19 fragments (CYFRA 21-1) were analyzed based on the phase of the menstrual cycle, the average levels of cytokeratin-19 fragments (CYFRA 21-1) obtained in the proliferative phase were significantly higher than the secretory phase in both endometriosis and non-endometriosis groups. This result is the same as that of Gjavotchanoff (2015). There is a thickened functional stratum in the proliferative phase compared to the secretory phase. Based on the IHC examination, many cytokeratins were identified in the functional stratum ( Stewart *et al.*, 2011). Functional cells that undergo apoptosis or over-proliferation will release cytokeratin-19 into the system. Endometriotic functional tissue shows hyper-immunoreactivity to CYFRA 21-1 ( Gjavotchanoff, 2015).

The best sensitivity and specificity values for cytokeratin-19 fragment (CYFRA 21-1) levels in spot urine were found in the proliferative phase where the sensitivity and specificity values were 89.5% and 88%, 9%, respectively, with a cutoff value of 5.024 ng/ml in the cytokeratin-19 fragment (CYFRA 21-1) level compared to the secretory phase and the overall value regardless of the cycle phase. This shows that the cycle phase is the predominant factor that must be considered because the cytokeratin-19 fragment (CYFRA 21-1) is a product of epithelial cells in which the intensity of epithelial cells in the endometrium and endometriotic cells is strongly influenced by cycle phase.

However, the cytokeratin-19 fragments (CYFRA 21-1) spot urine test, unable to describe its level throughout the day so it must be compared with urine creatinine ( Gjavotchanoff, 2015). The presence of dilutional factors and circadian rhythms will affect the consistency of the examination. The standard in measuring urine protein is 24-h urine protein due to fluctuating protein excretion. However, the 24-h urine collection has many problems, so an alternative method can be used, specifically the urine protein and creatinine ratio recommended by the National Kidney Foundation and Kidney Disease Outcomes Global Improving (KDIGO) ( National Kidney Foundation, 2022).

Chemical exposure assessment is a crucial aspect of public health and environmental monitoring. To determine the validity of a spot urine sample for this purpose, various parameters such as urinary creatinine concentrations, specific gravity, and osmolality are measured. These measurements help to adjust for dilution and report accurate analyte results. Among the methods used to account for dilution and report results, creatinine adjustment is the most widely accepted method. It involves dividing the analyte concentration (micrograms of analyte per liter of urine) by the creatinine concentration (grams of creatinine per liter of urine) and presenting the results as the weight of analyte per gram of creatinine (micrograms of analyte per gram of creatinine). This method is highly effective in ensuring accurate and reliable reporting of analyte levels in urine samples ( Barr *et al.*, 2005).

The urine creatinine ratio was calculated as a constant level for the validity of the results of the levels of cytokeratin-19 fragments (CYFRA 21-1) spot urine test. Based on the analysis of the cytokeratin-19 fragment (CYFRA 21-1) to creatinine (CYFRA/Cr) urine ratio, it was found that the mean in the endometriosis group was significantly higher than in the non-endometriotic group. Based on the cycle phase, it was found that the average cytokeratin-19 fragment (CYFRA 21-1) to creatinine (CYFRA/Cr) urine ratio was significantly higher in the proliferative phase than the secretory phase in both the endometriosis and non-endometriosis groups. Compared with the levels of cytokeratin-19 fragments (CYFRA 21-1) in spot urine when can be seen differences in the results of the analysis in the secretory phase. Creatinine has been used as a reference value for calculating ratio in the dilution of the sample and matrix ( Gjavotchanoff, 2015). Other similar studies that calculated the ratio of cytokeratin-19 fragments (CYFRA 21-1) to creatinine (CYFRA/Cr) urine ratio have not been found, so it is difficult to compare this study with others. No research has been found that analyzes the cutoff value of cytokeratin-19 fragments (CYFRA 21-1) to creatinine (CYFRA/Cr) urine ratio in endometriosis. A study by Lessey *et al.* (2015) and Gjavohanoff (2015) have applied creatinine as a determinant; however, in explanation, the results of cytokeratin-19 fragments (CYFRA 21-1) to creatinine (CYFRA/Cr) urine ratio were not delivered.

Based on recommendations from the National Kidney Foundation and Kidney Disease Outcomes Global Improving (KDIGO), the measurement of microprotein levels should be calculated based on urine creatinine levels as a stable matrix level in urine. This is because microproteins, which are one of the cytokeratin-19 fragments (CYFRA 21-1), are excreted in the urine at fluctuating levels, so it is not recommended to have a urine test during this time. For this reason, the measurement of cytokeratin-19 fragment (CYFRA 21-1) levels compared to urine creatinine as an alternative ( National Kidney Foundation, 2002).

In this study, the best sensitivity and specificity were found in the cytokeratin-19 fragments (CYFRA 21-1) to creatinine (CYFRA/Cr) urine ratio measured in the proliferative phase, which is even better than the measurement of cytokeratin-19 fragment (CYFRA 21-1) spot urine. It was difficult to compare the finding of this cutoff value with other studies due to the limitations of the study, which divides sensitivity and specificity values based on the cycle phase. The other limitation is cycle phase determination, which was based on the patient’s menstrual history, not on the results of endometrial curettage. Accurate phasing should look at the histopathological features of the endometrium.

CYFRA 21-1 spot urine levels are significantly higher in endometriosis than in non-endometriosis cases and are higher in the proliferative phase than in the secretory phase. The CYFRA/Cr ratio is also significantly higher in endometriosis than in non-endometriosis cases and higher in the proliferative than the secretory phase. Strengthening the accuracy of CYFRA 21-1 as a biomarker through correction of urine creatinine indicated by higher sensitivity and specificity of CYFRA/Cr than spot urine CYFRA, especially examined in the proliferative phase. It can be concluded that the CYFRA/Cr ratio can strengthen CYFRA 21-1 spot urine as a potential biomarker of endometriosis.

## Data Availability

figshare: CYFRA 21-1 strengthening using Urine Creatinine, https://doi.org/10.6084/m9.figshare.24085353.v1 ( Saputra *et al.*, 2023). This project contains the raw, underlying data. Data are available under the terms of the Creative Commons Attribution 4.0 International license (CC-BY 4.0).
