# Supplementary material for: Strengthening of CYFRA 21-1 using urine creatinine correction as potential endometriosis biomarker 
Source: F1000Res. 2025 May 14;13:46. Originally published 2024 Jan 8. [Version 2] doi: 10.12688/f1000research.135167.2 (PMC12134733; doi:10.12688/f1000research.135167.2)
Supplement: Peer review history

# Reviewer report: Reviewer response for version 2

**DOI:** 10.5256/f1000research.170068.r387639

**Published:** 2025-06-03

**Authors:** Daniel Vaiman

**Affiliations:** 1 Université de Paris, Paris, France

Endometriosis is a disease with a long period of absence of diagnostic (estimated at 7 years on average and at least). Having molecular markers proving the disease is thus an important issue for patients throughout the world.

 The study is based upon the urinary measure of the ratio CYFRA 21-1 / creatinine as a biomarker. CYFRA 21-1 is a fragment of cytokeratin n19

 The rationale is the following: the protein was identified initially in a small sample of 16 patients in urine, with reported variations according to the period of the cycle. Variations observed impede the use of the molecule as a markler, but here, the authors proposed to correct the level by the creatine level inside the urine. In the study 73 patients were included (38 endometriosis and 35 controls, each category being divided almost equally between proliferative and secretive phases).

 The result reveals excellent ROC curves, especially in the proliferative phase, and this is largely improved when the ratio is used, assuming an AUC of 0.99 and 0.95 in proliferative and secretive phases, respectively.

 This is an extremely nice result using a single marker. Discussion might remain to be written about the capability of this test to distinguish the patients at early stages of the disease, maybe before the symptoms reach high levels, or are very well established.

 It should be emphasized the care for the quality of the definitions of the patients, which while the number is relatively limited appears as a unusual effort in the literature in the field.

Is the work clearly and accurately presented and does it cite the current literature?

Yes

If applicable, is the statistical analysis and its interpretation appropriate?

Yes

Are all the source data underlying the results available to ensure full reproducibility?

Yes

Is the study design appropriate and is the work technically sound?

Yes

Are the conclusions drawn adequately supported by the results?

Yes

Are sufficient details of methods and analysis provided to allow replication by others?

Yes

Reviewer Expertise:

Reproductive Biology

I confirm that I have read this submission and believe that I have an appropriate level of expertise to confirm that it is of an acceptable scientific standard.

# Reviewer report: Reviewer response for version 1

**DOI:** 10.5256/f1000research.148270.r278758

**Published:** 2024-07-26

**Authors:** Ceylan ÖZSOY HEPOKUR

**Affiliations:** 1 Cumhuriyet University, Sivas, Turkey

The manuscript investigated whether CYFRA 21-1 is a biomarker in endometrosis patients. Three groups of patients were studied.

 The indexing of this article is acceptable with minor revision.

 1. Direct abbreviation is used in the title (CYFRA 21-1), it is recommended to write the open name to get easier citation.

 2. It is recommended to express how the number of patients was chosen statistically (p power analysis).

Is the work clearly and accurately presented and does it cite the current literature?

Yes

If applicable, is the statistical analysis and its interpretation appropriate?

Yes

Are all the source data underlying the results available to ensure full reproducibility?

Yes

Is the study design appropriate and is the work technically sound?

Yes

Are the conclusions drawn adequately supported by the results?

Yes

Are sufficient details of methods and analysis provided to allow replication by others?

Yes

Reviewer Expertise:

obstetrics and gynaecology, Cancer, mİRNA, circRNA

I confirm that I have read this submission and believe that I have an appropriate level of expertise to confirm that it is of an acceptable scientific standard.

# Response

**Published:** 2024-08-05

**Authors:** Hendy Hendarto

**Affiliations:** Obstetrics and Gynecology, Universitas Airlangga, Surabaya, East Java, Indonesia

Dear Reviewer,

 Thank you for reviewing our work.

 We hope it will give a valuable insight for the readers.

 Thank you again.

 Best regards

# Reviewer report: Reviewer response for version 1

**DOI:** 10.5256/f1000research.148270.r256952

**Published:** 2024-05-28

**Authors:** Simon M Scheck

**Affiliations:** 1 University of Otago, Dunedin, Otago, New Zealand

This paper presents a larger valdiation of a previously suggested urinary marker to detect endometriosis. The novel approach of correcting using urinary creatinine is also tested and validated.

 The methodology is overall well done and the results are promising and warrant publication to spark further research. I would like to recommend a few changes.

 The source data provided is incomplete - the creatinine levels and the endometriosis diagnosis is not included.

 There is very little clinical data provided about the patients - why they were undergoing surgery, how endometriosis was diagnosed, and the extent/stage of disease needs to be provided - especially relevant for application as a biomarker.

 The discussion needs some work - is very long but doesn't actually discuss the key results or implications of these results in enough detail.

 Specific comments:

 Introduction

 Paragraph 1: Citing specificity and sensitivity for laparoscopy vs histopathology – this is a small study from 2008 and no longer relevant – all current international guidelines accept laparoscopy as the gold standard of diagnosis – cannot compare laparoscopic techniques of 2024 with those of 2008

 Paragraph 3: “Cytokeratin-19 is found in simple and complex squamous epithelium” … “it is expressed in glandular-type epithelium” - squamous or glandular??

 Paragraph 3: “increasing serum creatinine levels that cause decreasing in the level of cytokeratin-19 fragments (CYFRA 21-1) in the urine accompanied the increasing its serum level in urine” – serum level in the urine does not make sense… either serum or urine?

 Paragraph 3: “The mean levels of cytokeratin-19 fragments (CYFRA 21-1) were significantly higher in the proliferative phase than in the secretory phase” – it is unclear what this refers to… ? Serum, urine, endometrial samples? Patients with endometriosis? Normal women?

 I think it’s important to point out that the Tokushige study of n=16 did mass spectrometry looking at 917 different “protein spots” and found that CK-19 was different – they did not hypothesize that this particular protein would be relevant – and in the small sample size and the large number of multiple comparisons, it is possible this was due to chance and therefore further large scale studies (such as this one are important)

 Methods

- What was the indication for laparoscopy? Infertility or pain? Did the “non endometriosis” patients include those undergoing laparoscopy for other causes, such as tubal ligation, hysterectomy, ovarian cysts, etc… How thoroughly were those patients screened for endometriosis at laparoscopy?
- How was endometriosis diagnosed? Laparoscopy or histology? Was any staging information recorded?  Do you have information about those with endometriomas or any other detail around the level of disease? (Small volume peritoneal disease may not be the same disease process as large volume nodular disease, endometriomas, etc.)
- “The AUC area at 80.7%” – AUC is not a percentage
- Could you present the data in table 2 and table 3 as box and whisker plots? Would be easier to interpret

Discussion

- Start the discussion with a summary of the key findings: in the proliferative phase this study shows a very high sensitivity, specificity and AUC for CYFRA 21-1:creatinine ratio for detecting endometriosis - and make this the focus of the discussion
- The discussion has a lot of discussion around possible biological explanations for CYFRA 21 being excreted in urine, but not much discussion of the actual results
- I would like to see a more detailed discussion around:
  - The results are highly significant in the proliferative phase without the creatinine correction – but they are improved with the creatinine correction – can you directly compare with and without creatinine correction and comment on this
  - The results in the secretory phase are less significant both with and without the creatinine correction - is it possible that normal endometrium creates CYFRA 21 in the secretory phase, but that endometriosis tissue creates CYFRA 21 at all times, making it a more useful marker in the secretory phase? Is there any data to suggest what would happen for women on menstrual suppression (e.g. progesterone treatment) which is common in endometriosis patients?

Is the work clearly and accurately presented and does it cite the current literature?

Yes

If applicable, is the statistical analysis and its interpretation appropriate?

I cannot comment. A qualified statistician is required.

Are all the source data underlying the results available to ensure full reproducibility?

Partly

Is the study design appropriate and is the work technically sound?

Yes

Are the conclusions drawn adequately supported by the results?

Yes

Are sufficient details of methods and analysis provided to allow replication by others?

Partly

Reviewer Expertise:

I am a gynaecologist with experience in treating patients with endometriosis medically and surgically. I have limited lab experience, but have been involved with studies looking at cervical, vaginal and blood based biomarkers for endoemetriosis.

I confirm that I have read this submission and believe that I have an appropriate level of expertise to confirm that it is of an acceptable scientific standard, however I have significant reservations, as outlined above.

# Response

**Published:** 2024-08-05

**Authors:** Hendy Hendarto

**Affiliations:** Obstetrics and Gynecology, Universitas Airlangga, Surabaya, East Java, Indonesia

Dear Reviewer,

 Thank you for reviewing our work. We managed to revise the article according to your comments and suggestion.

 1. We added more details on the creatinine level assessment and endometriosis diagnosis method.

 2. We added more details on the creatinine level assessment and endometriosis diagnosis method. The rASM data is not presented here as it will be published elsewhere.We added more details on the creatinine level assessment and endometriosis diagnosis method. The rASM data is not presented here as it will be published elsewhere.

 3. We added more data in introduction.

 4. Page 2 Line 42-43; 44-45

 Cytokeratin-19 is expressed in 2 epithelia. Commonly on the simple and complex squamous cell epithelium. However, on endometrium, it is expressed by endometrial gland epithelium.

 5. We revised the statement. The higher CYFRA 21-1 was in the urine of women with endometriosis.

 6. We added the discussion of Tokushige’s publication as suggested.

 7. We added more details on the sample selection.

 8. We added more details on the endometriosis diagnosis method.

 9. We revised the AUC value to decimal.

 10. We added the summary of findings in the start of discussion.

 11. We reduced the CYFRA-21 discussion as requested.

 12. The result is significantly difference and we only have the result comparison between CYFRA and CYFRA with creatine treatment as we showed in the manuscript.

 13. The growth of endometrial tissue in endometriosis causes an increase in the extent of functional tissue where the tissue contains cytokeratin-19 components. In endometriosis, progesterone resistance and increased estrogen occur which have an impact on increased cell proliferation and mitosis. Based on research by Kanaji et al 2011, the mechanism of cytokeratin release into extracellular is caused by apoptosis, disruption of cell membranes, cell division, surgical intervention, tissue necrosis caused by abnormal proliferation and this increases in the proliferation phase where the eutopic and ectopic endometrium experience abnormal proliferation caused by increased estrogen, where the nature of estrogen is proinflammatory and oncogenic. In contrast to the secretion phase where there is an increase in the hormone progesterone, Progesterone produced during the secretion phase can cause the endometrial mucosa not to be released, which means that endometriosis tissue also does not bleed and can develop into cysts. This explains why pregnancy, which increases progesterone levels, can reduce the development of endometriosis cysts. The nature of progesterone as an anti-inflammatory. Based on the Sperof book (2020) where stable endometrial conditions occur in estrogen and progesterone conditions. Where the endometrial wall is not fragile, there is no excessive proliferation.

 So it can be concluded that assessing endometriosis with urine cytokeratin 19 is best in the proliferation phase.

 Reference:

 Kanaji N, Bandoh S, Ishii T, Fujita J, Ishida T, Matsunaga T, Kubo A. Cytokeratins negatively regulate the invasive potential of lung cancer cell lines. Oncol Rep. 2011 Oct;26(4):763-8. doi: 10.3892/or.2011.1357. Epub 2011 Jun 23. PMID: 21701782.

 14. As we do not have any data from subjects with menstrual suppression so we cannot comment in this moment.

 Those are the responses to your comments and suggestions. We also upload the point-to-point responses.

 Thank you for your concern.

 Best regards
